# In this month’s Bulletin

**DOI:** 10.2471/BLT.21.000221

**Published:** 2021-02-01

In the editorial section, Viroj Tangcharoensathien et al. (78) present research on COVID-19 response and mitigation measures, the focus of this month’s issue and of the 2021 Prince Mahidol Award Conference.

In the news section, Gary Humphreys and Sophie Cousins report (81–82) on initiatives to reduce the environmental impact of the health sector. Poornima Prabhakaran talks to Andréia Azevedo Soares (83–84) about climate-related health hazards in India, initiatives to address them, and the challenges presented by industrial development. 

## China

### COVID-19, economic burden 

Huajie Jin et al. (112–124) estimate cost-of-illness.

## Singapore

### COVID-19, trust in government, and behaviour change

Vanessa W Lim et al. (92–101) report findings from cohort surveys.

## Zimbabwe

### How to engage on the COVID-19 response 

Constance RS Mackworth-Young et al. (85–91) analyse community perspectives.

## Global

### Who’s who on climate change?

Niheer Dasandi et al. (102–111) examine intergovermental engagement on health impacts of climate change.

### Which countries are measuring small particles?

Yevgen Nazarenko et al. (125–137) compare air quality standards.

### How do ethics committees work?

Johannes Köhler et al. (138–147) study the practices of national ethics and bioethics committees.

### A fresh start for policy?

Jennifer Cole and Klaus Dodds (148–154) ask if the COVID-19 response can reform climate change.

### Pros and cons of immunity certificates

Teck Chuan Voo et al. (155–161) review the ethical implications of immunity certificates in the context of COVID-19.

### Whose data?

Ann Dulhanty (162–163) discusses ethics of data collection and use.

### Infections and smoking

Freddy Sitas et al. (164–165) point to a need for better data and public information about the effects of smoking on outcomes of respiratory infections.

### Rounding out social determinants

Albert Persaud et al. (166–168) describe geopolitical factors affecting health.

